# Perspective taking in language: integrating the spatial and action domains

**DOI:** 10.3389/fnhum.2013.00577

**Published:** 2013-09-17

**Authors:** Madeleine E. L. Beveridge, Martin J. Pickering

**Affiliations:** Department of Psychology, University of EdinburghEdinburgh, UK

**Keywords:** embodied cognition, spatial perspective, agency, action perspective, situation models

## Abstract

Language is an inherently social behavior. In this paper, we bring together two research areas that typically occupy distinct sections of the literature: perspective taking in spatial language (whether people represent a scene from their own or a different spatial perspective), and perspective taking in action language (the extent to which they simulate an action as though they were performing that action). First, we note that vocabulary is used inconsistently across the spatial and action domains, and propose a more transparent vocabulary that will allow researchers to integrate action- and spatial-perspective taking. Second, we note that embodied theories of language comprehension often make the narrow assumption that understanding action descriptions involves adopting the perspective of an agent carrying out that action. We argue that comprehenders can adopt embodied action-perspectives other than that of the agent, including those of the patient or an observer. Third, we review evidence showing that perspective taking in spatial language is a flexible process. We argue that the flexibility of spatial-perspective taking provides a means for conversation partners engaged in dialogue to maximize similarity between their situation models. These situation models can then be used as the basis for action language simulations, in which language users adopt a particular action-perspective.

## Introduction

Over the past decade, research into language comprehension has increasingly been framed in terms of a link between perceptual and motor systems, and higher level cognitive tasks. A central assumption of such *Embodied Cognition* frameworks is that people’s understanding of language is grounded in their physical interactions with the world (e.g., Barsalou, 1999, 2008; Pulvermüller, 2005; Fischer and Zwaan, 2008; Glenberg et al., 2008a; Glenberg and Gallese, 2012). In strong versions of Embodied Cognition, language comprehension is achieved through mental representations that correspond, in perceptual or motor qualities, to the object or action being described. Such accounts draw on evidence that comprehenders are faster to correctly match sentences to images that correspond to the perceptual characteristics implied by the sentence context, such as orientation (Stanfield and Zwaan, 2001), shape (Zwaan et al., 2002; Pecher et al., 2009), and implied movement (Kaschak et al., 2005, 2006).

In addition, Action-Sentence Compatibility Effects (ACE; Glenberg and Kaschak, 2002) demonstrate that language comprehension is linked to action execution. Participants are faster to respond to sentences that imply moving the hand away from or towards one’s body (e.g., “Close/Open the drawer”), when the direction of response required (away from or towards their body) matches the direction of movement implied in the sentence. Aravena et al. (2010) recently provided evidence of a neural signature for ACE effects by recording event-related (brain) potentials. In this study, participants listened to sentences implying an open or closed hand shape, and indicated their understanding by responding with either an open or closed hand shape. Incongruent trials, where the hand-shape implied by the sentence did not match the hand-shape required by the response, resulted in an N400 effect (associated with difficulty integrating stimuli into a given semantic context; Kutas and Federmeier, 2000). Such evidence is consistent with the viewpoint that action language comprehension involves representing an action as though you were performing it yourself—that is, from an agent’s perspective.

In this paper, we explore research into action-perspective taking (from whose perspective do language users simulate a described action?), and spatial-perspective taking (from whose perspective do language users conceive spatial relations?). We propose that these two forms of perspective taking are fundamentally linked: in order for language users to perform an action simulation, they must first establish a spatial context for that action, by locating it within a situation model. In dialogue, spatial-perspective taking can be used by interlocutors to negotiate or align on situation models that specify similar spatial relations between entities, to ensure a mutually understood spatial context for actions. Actions are performed in space, and, therefore, we might expect considerable cross-over between the literatures on action- and spatial-perspective taking, but this does not appear to be the case. We argue that one reason for this situation is the use of inconsistent and conflicting terminology across the two fields. Our goal in this paper is to unite action- and spatial-perspective taking in an account of action language comprehension. First, we propose a vocabulary for discussing action-perspective taking that will allow action- and spatial-perspective taking to be integrated. Next, we explore evidence from the Embodied Cognition literature, investigating which action-perspective comprehenders typically adopt. We argue that, contrary to some Embodied Cognition accounts where action-perspective taking is typically assumed to be fixed on the agent, several other perspectives are in fact available. We then review research into which spatial-perspective people tend to adopt in language use, and how such perspective taking is negotiated in dialogue. Finally, we propose the Spatial Grounding Hypothesis, which states that action simulations are grounded in spatial context. We discuss the evidence in favor of this hypothesis, and explore the role of situation models in providing this context.

## Representing other people’s actions

At the same time as theories of action-language processing have stressed the primacy of motor representations, theories of action understanding have argued that the same mental representations are involved in both performing and perceiving actions (e.g., Grèzes and Decety, 2001; Prinz and Hommel, 2002). For example, Common Coding theory (Prinz, 1997; Hommel et al., 2001) proposes that codes for planned actions and perceived actions share a common representational domain. In support of this account, behavioral research suggests first, that participants are less able to perceive a static stimulus (left or right pointing arrow) when performing a congruent action (left or right button press; Müsseler and Hommel, 1997), and second, that perceiving an action while planning an incompatible action affects action execution (Brass et al., 2000; Kilner et al., 2003). In other words, the link between perception and action affects our ability both to *perceive* stimuli, and to *perform* actions. Such findings are echoed by recent neurological research showing evidence of “mirror matching”, where regions of the motor system that are activated when performing an action are also activated when passively perceiving an action (e.g., Buccino et al., 2001; Grèzes et al., 2003; for a review see Rizzolatti and Craighero, 2004).

Much research has argued that the perceiver of an action mentally simulates executing that action herself (Decety, 2002). This *simulation theory* has counterparts in simulation theories of mind that propose that understanding another person involves simulating their mental activity (e.g., Gallese and Goldman, 1998). Indeed, it could be argued that a successful theory of mind is one that allows us to predict and understand our own and other peoples’ actions, and that this is achieved through simulation (Ruby and Decety, 2001). The close link between self and other then begs the question: how do we distinguish our own actions or mental activities from those of other people? The ability to distinguish ourselves from other people is critical to successful social interaction, but in a system in which our own actions share representations with the actions of other people, action attribution becomes a key computational problem (Decety and Sommerville, 2003; de Vignemont and Haggard, 2008).

The mechanism by which the separation of self and other is maintained is beyond the scope of this paper (see, for example, Ruby and Decety, 2001, 2004; Decety and Sommerville, 2003). But however it is achieved, the self-other distinction is tightly connected with perspective taking. First, self must be successfully distinguished from other in order for there to be the possibility of different perspectives (Jeannerod, 2006). Second, the ability to represent other people’s actions in a similar way to their own allows people to take an agent’s perspective on an action, even when they are describing or hearing about an action performed by somebody else.

## A taxonomy of perspective

As highlighted above, a large body of research now suggests a link between language processing and sensorimotor activation (see Kiefer and Pulvermüller, 2012; Meteyard et al., 2012 for recent reviews). This link can best be captured by Embodied Cognition accounts of language processing^1^. Embodied Cognition seeks to distinguish itself from “traditional” psycholinguistic accounts by insisting that language representations are modal rather than amodal (e.g., Zwaan and Taylor, 2006; Barsalou, 2008). What is often not made explicit in Embodied Cognition accounts is that modal representations are inherently perspective-based. For a representation to be modal, it must assume a given perspective. In other words, the perspective is necessary to ground the representation. However, discussion of perspective taking in action language is often opaque, and this is particularly problematic if we wish to relate action-perspective taking and spatial-perspective taking.

In visual cognition, researchers distinguish between two types of spatial-perspective taking. Level 1 perspective involves understanding what falls within another individual’s line of sight—for example, is a particular object occluded by another object as that person looks at it? Level 2 perspective involves understanding how the world appears from another person’s perspective—for example, is a particular object to the left or the right of another object as that person looks at it? (Flavell et al., 1981; Michelon and Zacks, 2006). In the present paper, we limit our review of spatial-perspective to this second level, focusing on spatial relations, rather than visibility. Kessler and Rutherford (2010) argued that Level 2, but not Level 1 spatial-perspective taking, appears to involve some form of covert mental rotation or simulation. As such, Level 2 spatial-perspective entails a level of embodiment that Level 1 does not, and is therefore closer to the perspective-bound simulations proposed by Embodied Cognition accounts of action-language understanding.

With respect to Level 2 spatial-perspective taking, we can contrast intrinsic, absolute, and relative reference frames (see Levinson, 1996, 2003). In an intrinsic reference frame, the position of an object is described relative to a reference object (e.g., “The window is above the door”). In an absolute reference frame, the position of an object is described in terms of stable environmental features, such as points of the compass, as in “The ship is south of the island”. Neither of these reference frames locates an object relative to an observer. A relative reference frame, on the other hand, does just that: for example, “The car is to my left”. Within a relative reference frame, one can adopt an *egocentric* or *allocentric* perspective. An *egocentric* perspective entails representing objects in a scene from your own viewpoint, and an *allocentric* perspective entails representing objects from the viewpoint of someone other than yourself (see Levinson, 2003 for a fuller treatment of spatial reference frames). The terms *egocentric* and *allocentric* therefore have specific and well-established meanings in the spatial literature: *egocentric* means conceptualizing space from your own point of view, and *allocentric* means conceptualizing space from another’s point of view. In the literature on Embodied Cognition, however, researchers often use *egocentric* to refer to putting oneself in someone else’s shoes (for example, interpreting a sentence such as “John kicked Mary” as though the comprehender herself were performing the act of kicking; e.g., Willems et al., 2010). This use of the term is opposite that in spatial-perspective taking and is therefore confusing. In addition, using the term *egocentric* perspective in action language, or *allocentric* perspective in spatial language, does not specify *whose* shoes the comprehender is putting herself into. In spatial language, this underspecification is typically not problematic, since the perspective adopted in a sentence such as “John is looking at the picture on the left” can be explicitly clarified. The comprehender can legitimately ask “on whose left?”, and the speaker can reply “on *my* left”, “on *your* left”, “on *his* left”, etc. However, in action language, perspective-taking is implicit, rather than explicit, and no such clarification is possible. For example, a comprehender who responded to the sentence “John is looking at the picture on the left”, with the query “who is looking?” would receive the reply “John”, and remain no clearer about whose perspective the speaker was adopting. Therefore, unlike spatial language, when discussing action language it is necessary for embodied accounts to specify whose perspective is being adopted for a particular action: the term *egocentric* perspective tells us that comprehenders are putting themselves in somebody else’s shoes, but crucially not whose shoes. Similarly, researchers often speak of “situated simulations” (Marino et al., 2012), or “sensorimotor experience” (Pecher et al., 2009) without specifying from whose perspective this simulation or resonance occurs. We suggest that this lack of specification derives from a widely held assumption in embodied cognition accounts that the agent’s perspective is adopted. However, we also suggest that this assumption is unwarranted.

There are in fact different Embodied Cognition accounts of language processing, and researchers in this field place varying importance on the role of sensorimotor processing in semantics (see Meteyard et al., 2012 for a recent review of positions advocating different degrees of embodiment). However, a prevailing view conceives language comprehension as an internal simulation of the described action, as if the comprehender were performing that action herself (e.g., Barsalou, 1999; Zwaan and Taylor, 2006; Borghi and Scorolli, 2009; Bergen and Wheeler, 2010). If it is true that action-perspective taking is fixed on the agent’s perspective, then the underspecification of *egocentric*, outlined above, is not a problem; the perspective adopted would always coincide with the agent of the described action. However, as we shall see, it is not clear that an agent’s perspective is always adopted. Researchers in action language therefore need to make clear exactly whose perspective they assume is being adopted.

For example, in understanding “John kicked Mary”, there are at least two embodied perspectives that could be adopted for the action of kicking: that of John (the embodied agent); and that of Mary (the embodied patient). If the comprehender has reason to believe that other people are witnesses to the event (i.e., if she has reason to include bystanders in her situation model), then she can also adopt the perspective of a bystander watching the kicking event unfold (the embodied observer). For example, if a previous sentence implied the existence of a crowd gathering around Mary and John, the comprehender can adopt the perspective of a member of this crowd, observing John kicking Mary. In each case, the comprehender represents the action from the perspective of a person present in the comprehender’s model of that event. In taking the embodied agent’s perspective, the comprehender represents the action of kicking as though she herself were the agent of that action, by activating the same systems involved in executing a kicking action. In taking the embodied patient’s perspective, the comprehender represents the action of kicking as though she herself were the patient of that action (presumably activating some form of empathic response to the pain, such as wincing). In taking the embodied observer’s perspective, the comprehender represents that action as though she were watching it unfold, by activating the same systems that would be recruited when observing such an action. In addition to these embodied perspectives, there is another perspective that the comprehender could take: that of the non-embodied observer. Unlike an embodied participant or observer, the non-embodied observer represents the action without running a simulation from any particular point of view. We propose that action-perspective taking is grounded in spatial context (see section Situation Models: Linking Spatial- and Action-Perspectives); comprehenders will run an action simulation wherever possible, but if there is insufficient spatial context to simulate the action from a particular perspective, comprehenders will adopt the non-embodied observer’s perspective instead.

The sentence “John kicked Mary” refers to a transitive event with two participants. There are of course, more complex sentences in which further embodied perspectives exist. This is the case for sentences describing ditransitive events (e.g., “John passed the child to his wife”), or sentences where a thematic role is occupied by more than one entity (e.g., “John kicked Mary and Sam”). The number of potential embodied perspectives available for a given sentence is therefore the number of participants in that event plus that any embodied observers licensed by the comprehender’s situation model. We propose that these perspectives (e.g., embodied agent, embodied patient, embodied recipient, plus embodied observer and non-embodied observer) provide a transparent basis for discussing action perspective taking. Using these terms, researchers can not only distinguish between embodied and non-embodied representations, but within the embodied representations, it is possible to distinguish whose perspective is adopted.

## Do language users consistently adopt the agent’s perspective?

We noted above that many embodied accounts of language assume that if a perspective is adopted for action language, it is the agent’s perspective (e.g., Glenberg and Kaschak, 2002; Zwaan and Taylor, 2006; Wu and Barsalou, 2009). Such an assumption is consistent with results from studies using isolated action verbs, for example, showing somatotopic activation for specific body parts. Research using functional magnetic resonance imaging (fMRI) has found that passive listening to an arm-word (“pick”) leads to increased activation in areas of the premotor and primary motor cortex associated with arm movements; passive listening to a face-word (“lick”) leads to increased activation in areas associated with the face; and passive listening to a foot-word (“kick”) lead to increased activation in areas associated with the feet (Hauk et al., 2004; see also Aziz-Zadeh et al., 2006). In other words, the activation appears to be associated with particular acts from the perspective of the agent of the act (e.g., the kicker) rather than (for example) the patient (e.g., the person or thing that is kicked). Further work using magnetoencephalography (MEG) has demonstrated that such somatoptopic activation occurs extremely quickly, within 200 ms of word presentation, and even when participants are concentrating on an unrelated, non-language based task (Pulvermüller et al., 2005). These findings suggest that adopting an embodied agent’s perspective may occur automatically in the early stages of semantic processing, at least in isolated words^2^.

More evidence that people adopt the embodied agent’s perspective (as though the comprehender herself were carrying out an action) comes from evidence for “body-specific” representations of manual action verbs (e.g., *throw*) in a Dutch lexical decision task (Willems et al., 2010). Left-handed participants showed activation in the right pre-motor hand area, but right-handed participants showed activation in the left pre-motor hand area, despite there being no manual responses on critical trials. These results echo findings of “body-specific” activation for motor imagery, where left- and right-handed participants imagined performing actions described by manual action verbs (Willems et al., 2009). It therefore appears that people tend to adopt the embodied agent’s perspective for isolated verbs, representing the verb according to how they personally would perform those actions with their particular bodies (i.e., right-handed for right-handed participants; left-handed for left-handed participants).

However, verbs are usually processed not in isolation, but in the context of sentences featuring noun phrases that refer to particular entities. Do language users also adopt an embodied agent’s perspective in action sentences, as well as isolated verbs? The evidence that they do is mixed. Participants undergoing fMRI were presented with mouth-, leg-, or hand-related action sentences featuring the pronoun (“I”) in the agent’s role (e.g., “Mordo la mela” [I bite the apple]; “Afferro il coltello” [I grasp the knife]; “Calcio il pallone” [I kick the ball]; Tettamanti et al., 2005). The results showed evidence of somatotopic activation similar to that observed in isolated verb processing (e.g., Hauk et al., 2004), implying that participants were simulating the described actions from the agent’s perspective. However, in this study, the agent’s perspective coincided with the perspective of the potentially self-referential pronoun “I”: participants may have adopted a perspective in line with the thematic role assigned to the pronoun “I”, rather than the perspective of the agent *per se*. A better indication of whether participants routinely adopt the embodied agent’s perspective comes from studies investigating ACE effects (Glenberg and Kaschak, 2002; Glenberg et al., 2008b). When sentences were given in the form of an imperative (e.g., “Close the drawer”), participants were faster to respond when the direction of the response was congruent with the movement implied by the agent in the sentence than when it was incongruent. In other words, they appeared to adopt the perspective of an agent closing a drawer. However, in sentences featuring two arguments, one of whom could refer to the participant, participants were faster to respond when the direction of the response was congruent with the movement relative to the pronoun “you”. For example, participants were faster to respond with away movements to sentences such as “You delivered the pizza to Andy”, but faster to respond with towards movements to sentences such as “Andy delivered the pizza to you”. Therefore, this suggests that when a sentence involves a potentially self-referential pronoun (“you”, “I”), comprehenders tend to adopt the perspective of the thematic role assigned to that pronoun, whether or not this coincides with the thematic agent of the action. In a dialogue context, where sentences such as “You are / I am cutting the tomato” are uttered and understood by each participant in turn, the situation is more complex. Participants appear to prioritize adopting opposing perspectives for “you” and “I”, over maintaining a consistent perspective (e.g., embodied agent, embodied observer) for either of the pronouns (Pickering et al., 2012).

Several studies have addressed whether people adopt the agent’s perspective when the agent of a described action is not self-referential, in the absence of a second self-referential argument. In Embodied Cognition accounts that conceive action language as an extension of mirror-matching, where representations of other people’s actions are inherently similar to representations of one’s own actions (e.g., Rizzolatti and Arbib, 1998; Pulvermüller, 2005), descriptions of actions performed by third-person agents should elicit similar effects to descriptions of actions performed by first- or second-person agents. In line with this prediction, Buccino et al. (2005) used transcranial magnetic stimulation (TMS) to stimulate the left-hemispheric hand or foot motor areas, as participants listened to third person hand- or foot-related action sentences (e.g., “Cuciva la gonna” [He sewed the shirt]; “Marciava sul posto” [He marched on the spot]), compared with control abstract sentences (e.g., “Amava la moglie” [He loved his wife]). Motor evoked potentials (MEPs) from the hand and foot muscles were recorded. Hand MEPs were modulated specifically when listening to hand-related action sentences, and foot MEPs were modulated specifically when listening to foot-related sentences. These results suggest at least some tendency to adopt an embodied agent’s perspective for third-person sentences.

However, without a direct comparison between first- and third-person sentences, we cannot know whether action perspective-taking in third-person sentences matches action perspective-taking in first-person sentences. Behavioral evidence suggests that comprehenders reading self-referential and non-self-referential sentences adopt different action-perspectives. Brunyé et al. (2009) used a sentence-picture matching task with first-, second-, and third-person action sentences, and “internal” or “external” action images. In the “internal” images, the position of the hands meant they could plausibly be interpreted as those of the participant. In the “external” images, the position of the hands meant they could not plausibly be interpreted as those of the participant. Instead, they could most plausibly be interpreted as those of an agent who the participant was observing perform the action. Selecting an internal image would imply adopting the embodied agent’s perspective. Selecting an external image would imply adopting the perspective of an embodied observer. Brunyé et al. (2009) found that participants were faster to correctly match first- and second-person sentences to internal rather than external images, and to correctly match third-person sentences to external rather than internal images. In other words, participants adopted the embodied agent’s perspective when the agent of the sentence could be attributed to the comprehender, but not otherwise (see also Ditman et al., 2010; Sato and Bergen, 2013). In an fMRI study, Tomasino et al. (2007) found no difference in primary motor cortex activation between silent reading of German action phrases presented in the first-person (e.g., “Ich hämmere” [I hammer]) versus third-person (e.g.,“Er hämmert” [He hammers]). However, Papeo et al. (2011) had participants silently read action or non-action Italian verbs conjugated in the first- or third-person (e.g., “Scrivo” [I write]; “Scrive” [he writes]; “Medito” [I wonder]; “Medita” [he wonders]). They found that TMS-induced MEPs in the relevant motor area (e.g., hand) increased for the first-person action verbs, but that the third-person action verbs behaved like the non-action verbs, and showed no increase in MEPs. Embodied Cognition accounts need not predict total parity between first- and third-person action representations. However, the posited involvement of the motor system in action language comprehension (e.g., Fischer and Zwaan, 2008) should imply at least some difference between third-person action and non-action verbs. The fact that a difference between action and non-action verbs was found only in first-person sentences led Papeo et al. (2011) to conclude that motor simulation of an action sentence occurs only when the self is identified as the agent of the action.

What could be behind the conflicting results of Tomasino et al. (2007), and Papeo et al. (2011)? One important difference may be in the task. Participants in Tomasino et al.’s study were asked to decide whether a described event took place inside or outside a building, and thus could complete the task without paying attention to whether the verb was presented in the first- or third-person. On the other hand, Papeo et al. instructed participants to determine the syntactic subject of a phrase, thus focussing attention on the contrast between first- and third-person agents. Researchers are becoming increasingly aware of the role of task demands and context in studies of Embodied Cognition. The conflicting results here add to evidence suggesting that motor representations of action language may not be activated automatically, but depend on aspects of the task, including depth of processing (Sato et al., 2008), sentence tense (Bergen and Wheeler, 2010), and relevance to task goals (Hoedemaker and Gordon, 2013). Indeed, it is possible to view the emphasis on the agent’s perspective in action-language research, as a result of task demands. The link between action and language has typically been investigated by studying congruency effects when participants execute actions during sentence processing (Zwaan and Taylor, 2006; Taylor and Zwaan, 2008), after sentence processing (Glenberg and Kaschak, 2002; Glenberg et al., 2008b), or before sentence processing (Glenberg et al., 2008a). When the emphasis of the task is to execute an action, it is perhaps not surprising that results seem to indicate that participants adopt the agent perspective. Other paradigms in embodied approaches to language follow sentence processing with image presentation rather than action execution. For example, participants are typically faster and more accurate to recognize an image of an object when it is presented in the same orientation (vertical/horizontal) as implied by the preceding sentence (Stanfield and Zwaan, 2001; see also Zwaan et al., 2002; Pecher et al., 2009). The authors interpret these findings as evidence that comprehenders run visual simulations of an event (i.e., they adopt an embodied observer’s perspective). The perspective adopted by comprehender may therefore depend on the task used to investigate it. It may even be possible to use the task to prime participants to adopt a given action-perspective, although we know of no study that has investigated this possibility.

In summary, some Embodied Cognition accounts of action language assume that people adopt an embodied agent’s perspective when comprehending action language, based on an internal simulation of performing that action (Zwaan and Taylor, 2006; Barsalou, 2009). Moreover, strong Embodied Cognition accounts assume that the agent’s perspective is automatically activated, regardless of contextual factors such as the reference of the sentence, as determined, for example, by the subject pronoun (Pulvermüller, 2005; Pulvermüller et al., 2005). The evidence outlined above suggests that people do adopt the embodied agent’s perspective for isolated verbs, and for sentences in which a potentially self-referential pronoun (“you”, “I”) is specified as the agent (Hauk et al., 2004; Pulvermüller et al., 2005; Willems et al., 2010). However, when a self-referential pronoun occupies a thematic role other than agent, comprehenders appear to adopt the perspective of the thematic role assigned to that pronoun, and not the perspective of the agent (Glenberg and Kaschak, 2002). When a third party is specified as the agent of an action, and no self-referential pronoun is present, some evidence suggests that comprehenders adopt the embodied agent’s perspective (Buccino et al., 2005; Tomasino et al., 2007), whereas other evidence suggests that people adopt an embodied observer’s perspective (Brunyé et al., 2009; Papeo et al., 2011). Although more data are clearly needed in order to draw firm conclusions about which perspective comprehenders adopt under which circumstances, current data demonstrate that adopting an agent’s perspective is not the only possibility during action language comprehension. As a consequence, the underspecified terms *egocentric* or *internal* perspective should be avoided when discussing action-perspective taking. Instead, researchers in Embodied Cognition should seek to employ more transparent terms that specify *in whose shoes* the comprehender is placing herself (e.g., *embodied agent, embodied patient, embodied observer*).

## Spatial-perspective taking

So far, we have reviewed evidence examining whose action-perspective language users tend to adopt when processing action language sentences. However, language users can also adopt a range of spatial-perspectives during language production or comprehension. Of particular interest is whether people adopt an egocentric spatial-perspective (conceiving spatial relations from their own point of view), or an allocentric spatial-perspective (conceiving spatial relations from another’s point of view).

Schober (1993) asked participants to describe the location of objects, either alone, to an imaginary addressee, or when in the same room as a conversational partner. Participants were more likely to describe the location from the addressee’s point of view, using terms such “on your left”, than from their own point of view. Schober (1995) also found that speakers tended to adopt the addressee’s perspective in task requiring the speaker to identify particular objects to an addressee. Interestingly, participants in Schober (1993) who described objects to an imaginary addressee were *more* likely to use the addressee’s perspective than participants whose conversation partners were present. With an addressee absent and unable to provide feedback, it may be safer for the speaker to assume the addressee’s perspective as often as possible. Duran et al. (2011), using a virtual reality paradigm, also found that participants were more likely to adopt an allocentric spatial perspective when told that they were interacting with a virtual, rather than real partner. It appears that believing that their partner was real allowed participants to shift more of the burden of mutual comprehension to their partner. The tendency to shift responsibility for effective communication to a conversation partner may be stronger when, as in Duran et al.’s (2011) study, that partner is making a request rather than providing information. Yoon et al. (2012) found that speakers in a modified referential communication task were more likely to use allocentric perspective when requesting something from their partner compared with giving information to their partner. Since it is in speakers’ interests to ensure that their requests are successfully understood, it is sensible for listeners to assume that speakers will adopt an allocentric perspective when making that request.

The above results show that spatial-perspective taking, like action-perspective taking, is a flexible process. By changing the perspective they adopt, speakers or listeners can shift more or less of the burden of mutual comprehension on to their partner. Further research suggests that during dialogue, people may attempt to minimize not only their own effort, but the collective effort of both conversation partners, by obeying what Clark and Wilkes-Gibbs (1986) term the principle of *least collaborative effort*. Speakers and listeners often appear to adopt spatial perspectives in a way that maximizes the resources available. The principle of least collaborative effort appears to be adopted especially in cases where one partner is judged less able to complete the communication task (Schober and Brennan, 2003). For example, Mainwaring et al. (2009) found that speakers were more likely to use an (allocentric) addressee’s perspective when the addressee was under increased cognitive load. Schober (2009) studied what happens when, unbeknownst to the participants, one partner in a conversation has better spatial ability than another, as determined by mental rotation test results. Participants were paired into a director and a matcher, with no knowledge of their own or their partner’s results on the mental rotation tests. The matcher selected a target circle from an array, based on the director’s spatial descriptions. Low-ability directors were more likely to take their own (egocentric) perspective, while high-ability directors were more likely to take their partner’s (allocentric) perspective. Over the course of the experiment, high-ability directors who were paired with low-ability matchers increased their use of allocentric perspective, whereas low-ability directors who were paired with high-ability matchers decreased their use of allocentric perspective. Note that these opposite patterns of behavior between high- and low-ability directors is in itself reason to be cautious of basing our understanding of spatial perspective-taking in language on university students of (presumably) high cognitive ability.

We argue that this online adaptation to a partner’s ability to engage in the communicative task is compatible with conversation as conceived as a joint action (Clark, 1996; Sebanz et al., 2006; Gambi and Pickering, 2011). In the case of spatial perspective-taking, the perspective that people adopt appears to depend at least partly on the ability of their partner to engage in the task. In the next section, we argue that maximising the collective resources in this way allows conversation partners to establish coherent situation models in both partners. Once these situation models have been established, language users are in a position to adopt a particular action-perspective when performing mental simulations of actions. However, interlocutors do not adapt only their use of spatial-perspective within a relative reference frame; they also appear to adapt their choice of reference frame itself. Evidence that conversation partners align on their use of reference frame comes from studies using a confederate-priming paradigm. Watson et al. (2004) studied participants’ use of an intrinsic versus a relative reference frame. Participants were more likely to use an intrinsic reference frame after the confederate had used an intrinsic frame than after the confederate had used a relative reference frame. Importantly, Watson et al. found participants regularly switched between reference frames. Spatial-perspective taking in dialogue is therefore highly flexible in order to allow for maximal alignment and hence maximal similarity in situation models. Whether such alignment on situation models occurs as a result of automatic priming (e.g., Pickering and Garrod, 2004, 2006), or of negotiating common ground (e.g., Clark, 1996) is beyond the scope of this paper, but we assume both possibilities remain open.

## Situation models: linking spatial- and action-perspectives

Much research on Embodied Cognition can be traced back to studies of situation models in language processing (e.g., Johnson-Laird, 1983; Van Dijk and Kintsch, 1983). According to recent accounts, situation models are representations of specific situations described in language, where events are connected along five dimensions: space, time, protagonist, causality, and intentionality (Zwaan et al., 1995; for a review of situation models in language, see Zwaan and Radvansky, 1998). Evidence suggests it is the content of these models, rather than linguistic form of the language itself, which is typically retained in memory and integrated into updated models as comprehension continues (Sachs, 1967; Johnson-Laird and Stevenson, 1970). For example, Bransford et al. (1972) demonstrated that participants who read the sentence “Three turtles rested on a floating log, and a fish swan beneath them” frequently selected the linguistically different but situationally equivalent sentence “Three turtles rested on a floating log, and a fish swam beneath it” in a recognition test (see also Barclay, 1973; Honeck, 1973). Many modern studies in the Embodied Cognition literature have found similar effects when the focus is shifted to online rather than memory processes. For example, Borghi et al. (2004) found that participants were faster to verify items typically found inside a given object (e.g., “steering wheel”) following a preamble placing them inside that same object (e.g., “You are driving a car”) versus outside it (e.g., “You are refuelling a car”). They proposed that participants used a mental simulation grounded in modal representations (e.g., of being inside or outside a car), which then guides property verification (see also Kosslyn et al., 1978).

Such mental simulations are a defining feature of embodied theories of language, and differ from the situation models discussed in text or discourse processing in that they appear to capture online processing during language comprehension. Whereas situation models represent the integration of knowledge about events and situations into a coherent, existing framework, mental simulations are concerned with the online action-perspective taking about a particular act (see also Zwaan, 2008 for discussion of the differences). We propose that this “nesting” of action simulations within situation models is what links spatial- and action-perspective taking in language. In order for a comprehender to adopt an embodied perspective on an action, that action must be grounded in a spatial context. This spatial context is provided by the comprehender’s situation model. Situation models are conceived from a particular spatial perspective; in dialogue, conversation partners maximize their resources and align on spatial-perspective and reference frames, in order to ensure suitably similar situation models, for example by making use of the principle of least collaborative effort (Clark, 1996). Recall that situation models can specify events across a number of dimensions (space, time, causality, etc.; Zwaan et al., 1995). For our purposes, “suitably similar” situation models means that the situation models of both interlocutors specify the same protagonists in roughly the same spatial relations to one another.

The spatial relations between objects and people are a fundamental part of situation models (Tversky, 1991), and might be specified at various levels of granularity, from coarse grained, specifying only overall direction, to fine grained, specifying exact distances. We propose that the minimum information required in a situation model in order to run an action simulation is the participants in that action and some (coarse-grained) information about the spatial relations in which they stand. This allows comprehenders to establish the direction and perhaps rough distance in which an action occurs, and thus to simulate it, adopting a particular action-perspective. When a sentence is interpreted self-referentially (because it involves pronouns such as “you” or “I”—and perhaps also, although we know of no study demonstrating this—when it refers to the comprehender by name), the comprehender creates a situation model grounded in his or her own body; other participants in the action are by default conceived as located in front of the comprehender. For example, in Glenberg and Kaschak (2002), sentences such as “You delivered the pizza to Andy” elicited ACE effects because the direction of an action could be established (away from the comprehender’s body), and an action-perspective could be adopted in line with the thematic role assigned to the self-referential pronoun (embodied agent). We refer to the idea that spatial context grounds action-perspective taking as the Spatial Grounding Hypothesis.

The Spatial Grounding Hypothesis can explain the diverging results we discussed earlier regarding first-person and third-person language. Recall that Papeo et al. (2011) found that comprehenders appeared to adopt an embodied agent’s perspective for first-person language, but no embodied perspective for third-person language; whereas the results of Tomasino et al. (2007) suggested that first- and third-person language elicited similar action perspectives. The Spatial Grounding Hypothesis explains these results as follows. In Papeo’s study, the first-person sentences ground the situation model in the comprehender’s own body, allowing an action simulation to occur; in the third-person sentences, the situation model contains insufficient spatial information for action simulation. In Tomasino et al.’s (2007) study, the task was to decide whether the described action took place inside or outside, thus encouraging the construction of situation models in which to situate first- *and* third-person actions. Task demands may therefore play an important role in action language understanding, in the extent to which they provide, or encourage participants to create, spatial context for the described actions.

For example, third-person sentences in which the direction of the described action (e.g., turning a knob clockwise or anti-clockwise) is apparent from the sentence context (e.g., raising or lowering the volume) also elicit ACE-type effects where the comprehender adopts an embodied agent’s perspective (Zwaan and Taylor, 2006). Further work suggests that these effects only occur once the direction of movement (clockwise or anti-clockwise) has been specified (Taylor et al., 2008). On the other hand, some evidence suggests that where a described action lacks suitable spatial grounding—for example, when it is described in the third-person, and the spatial relations between participants are not specified—action-perspective taking does not occur. Gianelli et al. (2011) replicated the ACE effects in sentences featuring second-person agents (e.g., “You gave a pizza to Louis”), but not third-person agents (e.g., “Lea gave a pizza to Louis”). When avatars provided spatial locations for the third-person agents, the ACE effect reappeared. In other words, participants only adopted an embodied agent’s action-perspective when their situation model afforded adequate spatial context.

We have suggested that spatial context grounds action-perspective taking, such that a comprehender can only simulate an action from a particular perspective if her situation model specifies the participants in that action, and their spatial relations (thus giving her access to the direction in which an action would occur). We have argued that this proposal, the Spatial Grounding Hypothesis, can incorporate apparently conflicting results about action-perspective taking into a coherent framework. But there are other factors that support the Spatial-Grounding Hypothesis. First, it predicts that conversation partners will align on spatial-perspective and choice of reference frame, in order to establish similar situation models in both partners. We saw in the previous section that this is indeed the case. Second, it can explain why the presence of a potential agent other than the speaker affects how likely the speaker is to shift her spatial perspective. Tversky and Hard (2009) investigated the influence of a potential agent on how likely people were to adopt an allocentric perspective. Participants viewed photographs of scenes in which an actor was reaching for objects (and thus, in a position to act on that object), scenes with no actor, and scenes with an actor who was not reaching. Participants were more likely to adopt an allocentric spatial perspective (that of the actor in the photograph) when the actor was reaching versus not reaching for an object. Similarly, Zwickel (2009) investigated what spatial-perspective participants adopted when watching clips of animated triangles that they perceived as more or less agentive (Abell et al., 2000). Zwickel provided some evidence that participants only adopt an allocentric perspective when they view the other entity as an agent with specific states of mind, rather than a non-agentive entity moving at random. Mazzarella et al. (2012) recently extended Tversky and Hard’s (2009) study by manipulating the extent to which the actor was in a position to act on the object (grasping versus gazing). Images in which the actor was in a better position to act on the object (grasping) triggered more use of allocentric spatial perspective in participants compared with images in which the actor was in a less good position to act on the object (gazing). All of this suggests that participants are more likely to adopt an allocentric spatial-perspective in the presence of someone they perceive as a potential agent.

On the other hand, research suggests that the ability to extract information useful for object interaction (e.g., size) is diminished when participants adopt an allocentric, rather than egocentric spatial-perspective (Campanella et al., 2011). In addition, participants are faster to execute a reach-to-grasp movement when the object also falls within the peripersonal, rather than extrapersonal, space of a second person, implying that people tend to be faster to interact with objects in the presence of another potential agent (Gianelli et al., 2013). Given that participants want to interact with objects more quickly in the presence of another potential agent, and given that adopting an allocentric perspective may impede their ability to do so, why, then, would participants be more likely to adopt an allocentric perspective in the presence of another potential agent? Tversky and Hard (2009) suggested that their participants, in order to make sense of the scene, tried to understand the possibility that the other person can interact with the objects. We propose that people find it easier to understand another person’s potential actions when they understand the spatial relations in the other person’s situation model; that is, when they conceive space from that person’s perspective. Spatial-perspective taking can therefore augment a situation model by increasing awareness of an agent’s *potential* actions, even when no action is described.

One argument against the Spatial Grounding Hypothesis is that that situation models are often underspecified, and do not provide comprehenders with the necessary spatial context in which to situation action simulations. In particular, isolated verbs provide no explicit spatial context, and yet evidence suggests that comprehenders do adopt an embodied agent’s perspective on the actions that the verbs describe (e.g., Hauk et al., 2004; Willems et al., 2010). We suggest that participants typically interpret these isolated verbs as self-referential (even when they are not presented in the imperative). Thus, like explicitly self-referential language, the comprehender’s own body grounds her situation model in this case. In other cases, where the comprehender’s situation model does not allow her to establish at least the coarsely-coded spatial relations involved in an action, she cannot adopt an embodied action-perspective, because the action simulation cannot be run. However, this does not mean that the sentence describing an action cannot be understood. Rather, the comprehender can adopt the perspective of a non-embodied observer. This perspective is not an embodied perspective, in the sense that it does not involve a simulation of the action from the perspective of any of the participants. However, it is sufficient to allow the comprehender to understand the sentence, even if that understanding is somewhat less fully specified than the situation in which an embodied action-perspective can be adopted. Researchers have found that non-ice hockey players respond more slowly and show less pre-motor activation than expert ice hockey players do when reading sentences about ice hockey (Beilock et al., 2008), but this does not mean that they fail to understand the sentences. Their understanding may be impoverished relative to that of the expert players, but comprehension is not an all or nothing process (Taylor and Zwaan, 2013). Just as non-expert players may supplement their understanding of ice hockey using information and inferences about similar experiences (e.g., playing field hockey), comprehenders with inadequate situation models may supplement their models by adopting a non-embodied observer’s perspective based on memories or inferences about similar situations.

## Conclusions

In this paper, we have attempted to reconcile two largely distinct literatures concerned with spatial-perspective taking and action-perspective taking. We have proposed a transparent vocabulary for action-perspective taking, which we hope will facilitate research between these two domains. At the heart of our proposal is the suggestion that researchers working in Embodied Cognition must specify *from whose perspective* a given action is being simulated. Although an agent’s perspective seems in many cases the most natural candidate, other perspectives are possible, and are often adopted when self-referential pronouns are assigned a thematic role other than agent.

We have argued that comprehenders can only adopt an action-perspective if they have a spatial context for that action (the Spatial Grounding Hypothesis). In the case of isolated verbs and self-referential pronouns, people typically take their spatial grounding from their own bodies. But in the absence of self-referential language, action-perspective taking can only occur when the spatial relations between participants in the action have been established within the comprehender’s situation model. In dialogue, interlocutors use spatial-perspective taking to ensure that each partner’s situation model specifies similar spatial relations.

## Conflict of interest statement

The authors declare that the research was conducted in the absence of any commercial or financial relationships that could be construed as a potential conflict of interest.

## Footnotes

^1^Note our use of the term “embodied” refers specifically to Embodied (or “grounded”) Cognition accounts of language, not to “embodied” versus “disembodied” perspectives, as sometimes discussed in the spatial perspective literature (e.g., Tversky and Hard, 2009).

^2^A general note of caution is needed when interpreting studies that show similar activation in action execution and action language comprehension. These studies are typically cited as evidence that during language comprehension, participants simulate performing the action (in our terminology, they adopt an embodied agent’s perspective). However, research into mirror-matching suggests that observing and executing an action also activate similar neural substrates (e.g., Grèzes et al., 2003; for reviews, see Decety and Sommerville, 2003; Rizzolatti and Craighero, 2004). Therefore, it is possible that activation in motor areas during language comprehension in fact reflects the participant mentally “observing”, rather than “executing”, the described action.
